# Efficacy of fluralaner plus moxidectin (Bravecto® Plus spot-on solution for cats) against *Otodectes cynotis* infestations in cats

**DOI:** 10.1186/s13071-018-3167-z

**Published:** 2018-11-19

**Authors:** Janina Taenzler, Christa de Vos, Rainer K. A. Roepke, Anja R. Heckeroth

**Affiliations:** 1 MSD Animal Health Innovation GmbH, Research Antiparasitics, Zur Propstei, 55270 Schwabenheim, Germany; 2 Clinvet, Uitsig Road, Bainsvlei, Bloemfontein, 9338 South Africa

**Keywords:** Bravecto Plus, Cat, Ceruminous exudate, Ear mite, Fluralaner, Isoxazoline, Miticide, Otitis externa, *Otodectes cynotis*, Otocariosis, Spot-on, Topical

## Abstract

**Background:**

The efficacy of the fixed combination of fluralaner plus moxidectin for the treatment of *Otodectes cynotis* infestations was evaluated in cats after topical application.

**Methods:**

Sixteen cats experimentally infested with *O. cyn*otis were allocated randomly to two groups of 8 cats each. One group was treated topically with the fixed combination of fluralaner plus moxidectin at the minimum dose rate of 40 mg fluralaner and 2 mg moxidectin/kg body weight. The other group was treated with physiological saline solution. Before and 14 and 28 days after treatment the ears of all cats were examined otoscopically for live mites and for the amount of debris and cerumen. Twenty-eight days after treatment, the cats were sedated and had both ears flushed to obtain the total number of live mites per animal. Efficacy was calculated, based on the results of the ear flushing, by comparing mean live mite counts in the fluralaner plus moxidectin treated group *versus* the saline group.

**Results:**

A single topical application of the fixed combination of fluralaner plus moxidectin to cats reduced the mean mite counts by 100% (*P* < 0.001) by 28 days after treatment. No mites were visible during otoscopic examination at either 14 or 28 days after treatment. All fluralaner plus moxidectin treated cats had less ceruminous exudate 28 days after treatment compared to pre-treatment and 14 days after treatment. No treatment related adverse events were observed in any cats enrolled in the study.

**Conclusions:**

Single topical application of the fixed combination of fluralaner plus moxidectin was highly effective against *O. cynotis* infestations in cats.

## Background

The ear mite, *Otodectes cynotis*, is one of the most common ectoparasites of cats in Europe and is considered to be the primary factor in more than half of the cases of otitis externa [1, 2]. It is more likely to be pathogenic in young cats and most likely directly transmitted from one animal to another [3]. Most often infestations in cats are only recognized by owners when intense pruritus accompanies the infestation. During clinical examination of the ear canal, erythema and dark brown, coffee ground-like, ceruminous exudate are characteristic findings in infected animals [3, 4]. Occasionally, the infestation leads to intense irritation and secondary bacterial infection, possibly resulting in purulent otitis externa [5].

Several treatments, mainly spot-on formulations of macrocyclic lactones for topical application containing the milbemycin moxidectin or the avermectin selamectin, are available for cats. These formulations provide an attractive alternative to the application of the avermectin ivermectin directly into the external ear canal (aural or ototopical treatment) because of the ease of application and monthly dosing regimen [6, 7].

Systemically active ectoparasiticides of the isoxazoline family have demonstrated miticidal efficacy. The efficacy of the spot-on formulation of sarolaner plus selamectin in the treatment of *O. cynotis* in cats has been shown by Becskei et al. [8]*.* Single oral treatment with afoxolaner of naturally infested cats demonstrated 100% efficacy over a period of 35 days [9]. This is the first study, which describes the use of afoxolaner for the treatment of otodectic mange in cats, to date afoxolaner is only registered for dogs. Of the isoxazoline class, sarolaner is the only member in combination with selamectin, which is registered for the treatment of *Otodectes* mites in cats, with a re-treatment interval of 4 weeks. The efficacy of fluralaner as a single agent, with a 3 months re-treatment interval, against *Demodex*, *Sarcoptes* and *Otodectes* mites in dogs [10–12] and *Otodectes* mites in cats [12] has been reported previously.

In order to provide an easy to use, broad spectrum ecto- and endoparasite treatment option for cats, moxidectin has been added to fluralaner in a new spot-on formulation to broaden the spectrum of activity to include the prevention of heartworm disease and treatment of gastrointestinal nematodes in cats. In the present study, the efficacy and safety of this new fixed combination product was evaluated in the treatment of experimental infestation with the ear mite *O. cynotis* in cats.

## Methods

The same methodology as already described by Taenzler et al. [12] was applied to investigate the miticidal efficacy of the fixed combination of fluralaner plus moxidectin for the treatment of *Otodectes cynotis* infestations after topical application in cats and, therefore, only differences with regards to methods and study set-up described by Taenzler et al. [12] are given below in detail.

Sixteen European mixed breed (short hair) cats, of both sexes (7 males castrated, 9 females intact), between 1 and 7 years old, weighing between 2.3 and 4.3 kg on the day of treatment, healthy upon physical examination, with a positive *O. cynotis* mite infestation, were randomly allocated to two study groups of 8 cats each.

Cats in the treatment group were treated once topically with fluralaner plus moxidectin spot-on solution at the minimum recommended dose rate of 40 mg fluralaner plus 2 mg moxidectin/kg body weight at the base of the skull. To maintain masking, cats in the control group were treated once topically with physiological saline solution (sodium chloride 0.9%). There was no evidence of dosing errors, such as spillage or run- or drip-off, in any of the treated cats. Depending on their individual body weight, cats in the treatment group had 0.33–0.57 ml fluralaner plus moxidectin and control cats had 0.35–0.62 ml physiological saline applied topically.

Before and 14 and 28 days after treatment, an otoscopic examination of both ears from each cat was performed and the results clustered into 4 categories. Additionally, on the same time points the amount of debris and cerumen was recorded. Twenty-eight days after treatment, each cat was sedated and both ears were flushed to determine the number of live mites. The percentage of efficacy against *O. cynotis* mites was calculated, using geometric means live mite counts in the fluralaner plus moxidectin treated group *versus* the saline group employing Abbott’s formula.

## Results

The treatment was well tolerated and no adverse events related to the topical application of the fixed combination of fluralaner plus moxidectin were observed in any of the cats.

All 16 cats had an adequate mite infestation in both ears. Additionally, all cats presented debris and/or cerumen in the external ear canals and were judged to be appropriate infested to be included in the study.

**Fig. 1:**
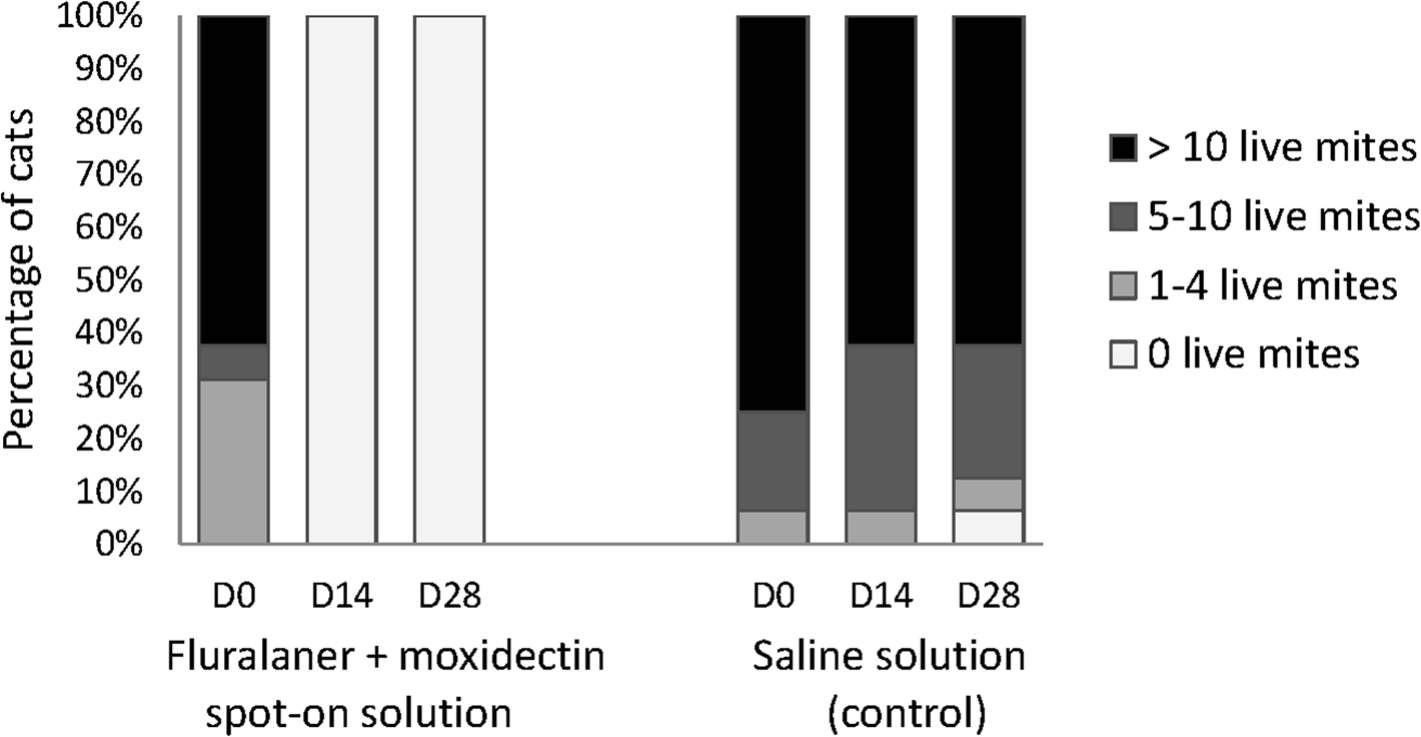
Cats with visible live mites observed during otoscopic examinations before and 14 and 28 days after treatment

**Fig. 2:**
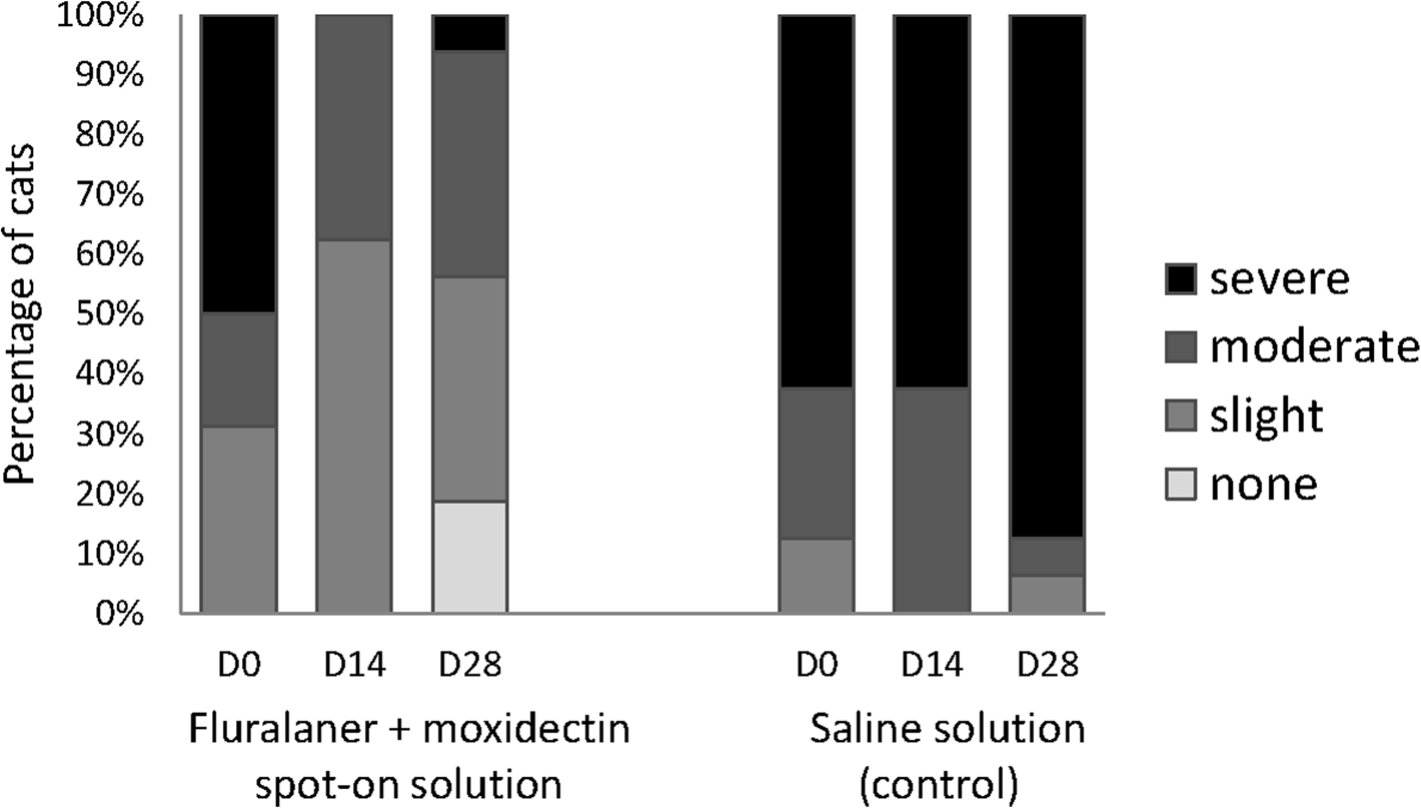
Cats with cerumen/debris observed during otoscopic examinations before and 14 and 28 days after treatment

After ear flushing 28 days after treatment, no mites were observed in either ear of any of the cats treated with fluralaner plus moxidectin, whereas a mean of 595.1 (range 6–1843) live mites was recorded in the control cats. This is a significant (*P* < 0.0059) reduction in mite count following treatment and 100% efficacy (Table 1). During otoscopic examination at both 14 and 28 days after treatment, no mites were visible in the fluralaner plus moxidectin-treated cats (Fig. 1). There was less cerumen and/or debris in the external ear canals of fluralaner plus moxidectin treated cats 28 days after treatment compared to pre-treatment and 14 days after treatment (Fig. 2).

**Table 1:** Mite counts and corresponding efficacy (%) of fluralaner plus moxidectin administered once topically against infestations with *O. cynotis* at 28 days after treatment

	Range (*n*)	Mean (*n*)^a^	Efficacy (%)
Fluralaner plus moxidectin treatment	0	0	100 (*P* < 0.0059^b^)
Negative control	6–1843	595.1	na

*Abbreviation*: *na* not applicable

^a^Arithmetic mean

^b^One-way ANOVA with a treatment effect (*F*_(2,21)_ = 58.44)

## Discussion

Topical application of the spot-on formulation fluralaner plus moxidectin completely eliminated ear mites from infested cats. The immediate decrease in the number of mites, shown by the absence of mites during otoscopic examination at 14 days after treatment, was observed together with the improvement of clinical signs of external ear disease (otitis externa) in these cats.

The life-cycle of *Otodectes cynotis* from egg to egg has a duration of 18–28 days, before the next generation of larvae, nymphs and adult mites develop [13]. In addition to the complete elimination of mites from the external ear canal, improvement in clinical signs is also very important to cat owners and veterinarians. Existing commercially available topical products for the treatment of *O. cynotis* infestation in cats either consist of one (selamectin) or a combination of two (imidacloprid and moxidectin; selamectin and sarolaner) active ingredients. Efficacy of these topical products over four weeks has been reported following either a single topical application [6, 14] or two applications one month apart [14, 15]. The efficacies, assessed in these studies one month after treatment, are in line with the results of the present study after single topical treatment with fluralaner plus moxidectin. [13]. The spot-on formulation of fluralaner plus moxidectin is an extended duration product with efficacy of up to 12 weeks [16]; however, the study observation period in the present study was 28 days (i.e. one mite generation). It is assumed that the efficacy of fluralaner plus moxidectin will extend beyond 28 days and that otic signs will continue to improve further, until fully resolved. Thus, a single application of fluralaner plus moxidectin not only has an effect on the existing mite population, but also disrupts the mite life-cycle and eliminates mites from the external ear canals of treated cats.

Co-infestations with several parasites are common in cats. Co-infestation with endoparasites and ectoparasites was found in 14% of 1519 cats, and 12% of this large group of cats had both ectoparasites (e.g. fleas or ear mites) and gastrointestinal helminths [2]. The combination of fluralaner plus moxidectin in a spot-on formulation with a 12-week treatment interval provides cat owners the opportunity to eliminate common co-infestations with ectoparasites and nematodes. The present study demonstrated the safety and efficacy of this novel spot-on formulation of fluralaner plus moxidectin (at a minimum dose of 2 mg/kg bodyweight) against *O. cyntotis* infestations in cats. The extended duration of activity of this novel product may help to promote owner compliance and aligns with veterinary treatment guidelines for endoparasite treatment of cats.

## Conclusions

A single topical application of the fixed combination of fluralaner plus moxidectin was highly effective in eliminating ear mite infestations in cats. A single topical application of fluralaner plus moxidectin treatment resulted in improvement of clinical signs of external ear disease (otitis externa) during the 28 day observation period. The treatment was well tolerated and safe in cats.
